# Atrioesophageal Fistula Following Atrial Fibrillation Ablation: A Case Report

**DOI:** 10.7759/cureus.94193

**Published:** 2025-10-09

**Authors:** Vivien Bogaert, Veronica Lorenz

**Affiliations:** 1 Cardiovascular and Thoracic Surgery Department, Cliniques Universitaires Saint-Luc, Brussels, BEL

**Keywords:** atrial fibrillation ablation, atrioesophageal fistula, cardiac surgery, mediastinitis, neurological complications

## Abstract

Atrioesophageal fistula (AEF) is a rare but catastrophic complication of atrial fibrillation (AF) ablation, with mortality exceeding 90% in the absence of surgical intervention. We report the case of a 68-year-old man who developed AEF four weeks after radiofrequency ablation for AF. The patient initially presented with pneumopericardium and retrosternal chest pain, progressing to mediastinitis. Surgical repair with esophageal suture and muscle flap interposition was performed. Despite initial improvement, the patient developed neurological complications following early resumption of oral feeding. A second operation revealed left superior pulmonary vein perforation and dehiscence of esophageal sutures, requiring esophagectomy with cervical esophagostomy. Cultures grew *Streptococcus mitis*, *Neisseria macacae*, and *Staphylococcus epidermidis*. After six weeks of intravenous antibiotics and full neurological recovery, the patient was discharged. This case highlights the diagnostic challenges of AEF, the critical role of contrast-enhanced chest CT, and the need for delayed oral feeding after surgical repair.

## Introduction

Catheter and surgical ablation procedures for atrial fibrillation (AF) can lead to rare but life-threatening complications, including atrioesophageal fistula (AEF) [1]. This condition may occur irrespective of the energy source used, including radiofrequency, cryoablation, and high-intensity focused ultrasound. Indeed, the use of these energy sources may result in thermal injury to adjacent structures, particularly the esophagus, which is located immediately posterior to the left atrium. Symptoms generally appear within two to four weeks after the procedure, with a median onset at 21 days [2]. The classic presentation includes fever and neurological deficits caused by cerebral air embolism, often associated with gastrointestinal bleeding, chest pain, or dysphagia [3]. Mortality is reported to be 97% without intervention, 65% with endoscopic management, and 33% after urgent surgical repair. We present a case of AEF following radiofrequency ablation of AF with an atypical presentation and delayed diagnosis.

## Case presentation

**Figure 1:**
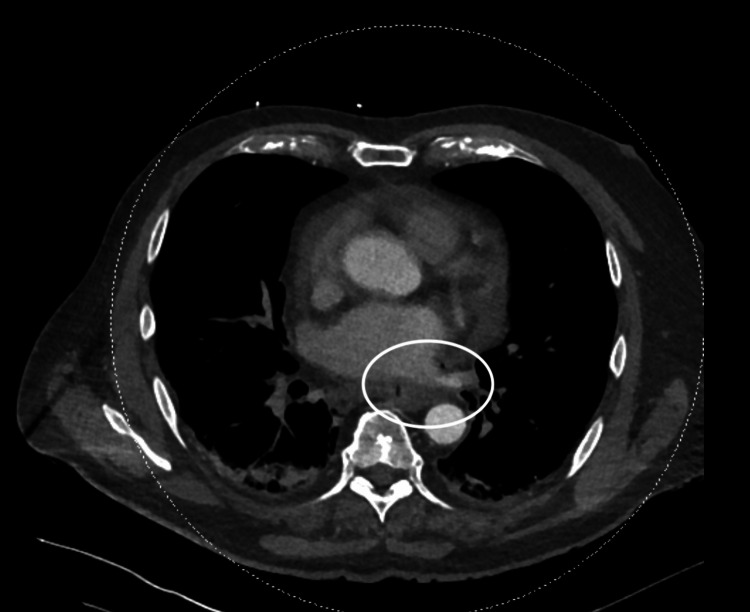
Computed tomography angiography showing pneumomediastinum

**Figure 2:**
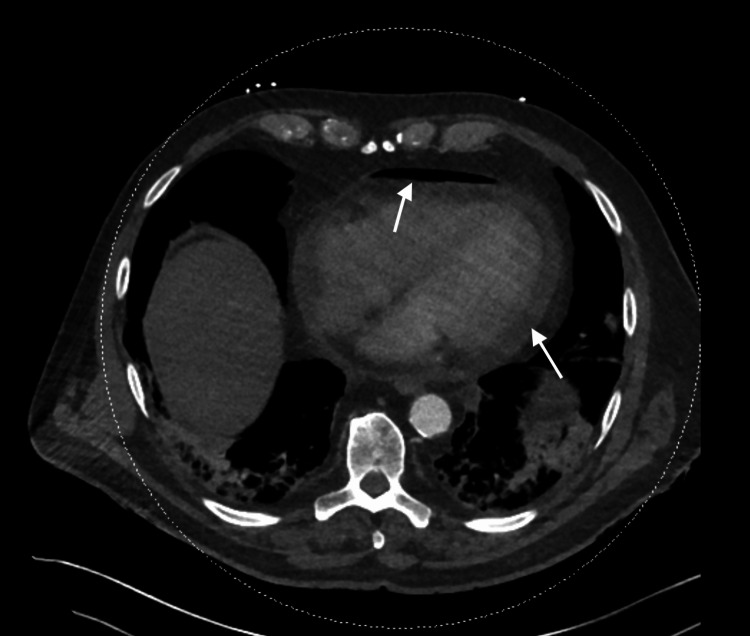
Computed tomography angiography showing pneumopericardium and pericardial effusion

**Figure 3:**
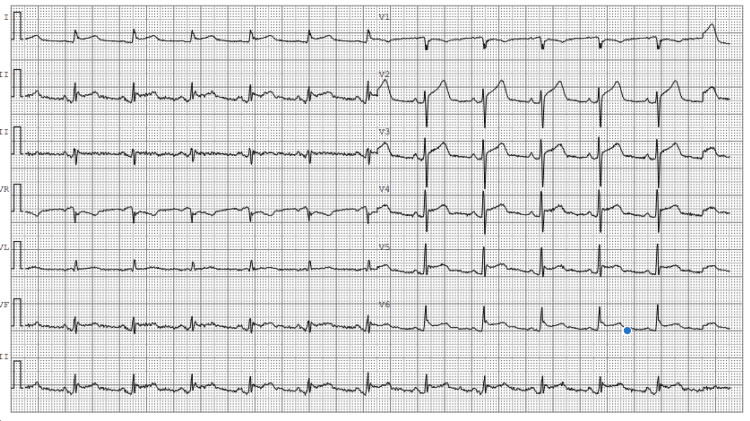
Electrocardiogram showing diffuse ST-segment elevation

A 68-year-old man with a history of atrial fibrillation underwent radiofrequency ablation four weeks before admission. He was referred to our hospital for pneumopericardium detected on imaging. The patient reported new-onset retrosternal chest pain at rest radiating to the jaw. Laboratory testing revealed elevated inflammatory markers with a C-reactive protein (CRP) of 250 mg/dL. Surprisingly, he has no fever. His chest CT scan demonstrated pneumomediastinum and pneumopericardium with pericardial effusion but no evidence of aortic dissection (Figures 1-2). The electrocardiogram showed diffuse ST-segment elevation (Figure 3).

Operative findings and initial management

A median sternotomy was performed, revealing a purulent pericardial effusion consistent with mediastinitis. The pericardial cavity was irrigated. Cardiopulmonary bypass (CPB) with aortic cross-clamping was established. Intraoperative endoscopy identified a 1-cm esophageal perforation located 30 cm from the dental arcade. Pericardial exploration revealed perforation adjacent to the left inferior pulmonary vein. A left atriotomy through Sondergaard’s groove showed no atrial wall perforation.

Direct esophageal repair was carried out, reinforced with an intercostal muscle flap interposition between the esophagus and pericardium [4]. A jejunostomy tube was placed. Postoperatively, the patient was treated with intravenous cefuroxime, metronidazole, and proton pump inhibitors. He remained in intensive care for four days and showed clinical improvement.

Clinical course and reoperation

**Figure 4:**
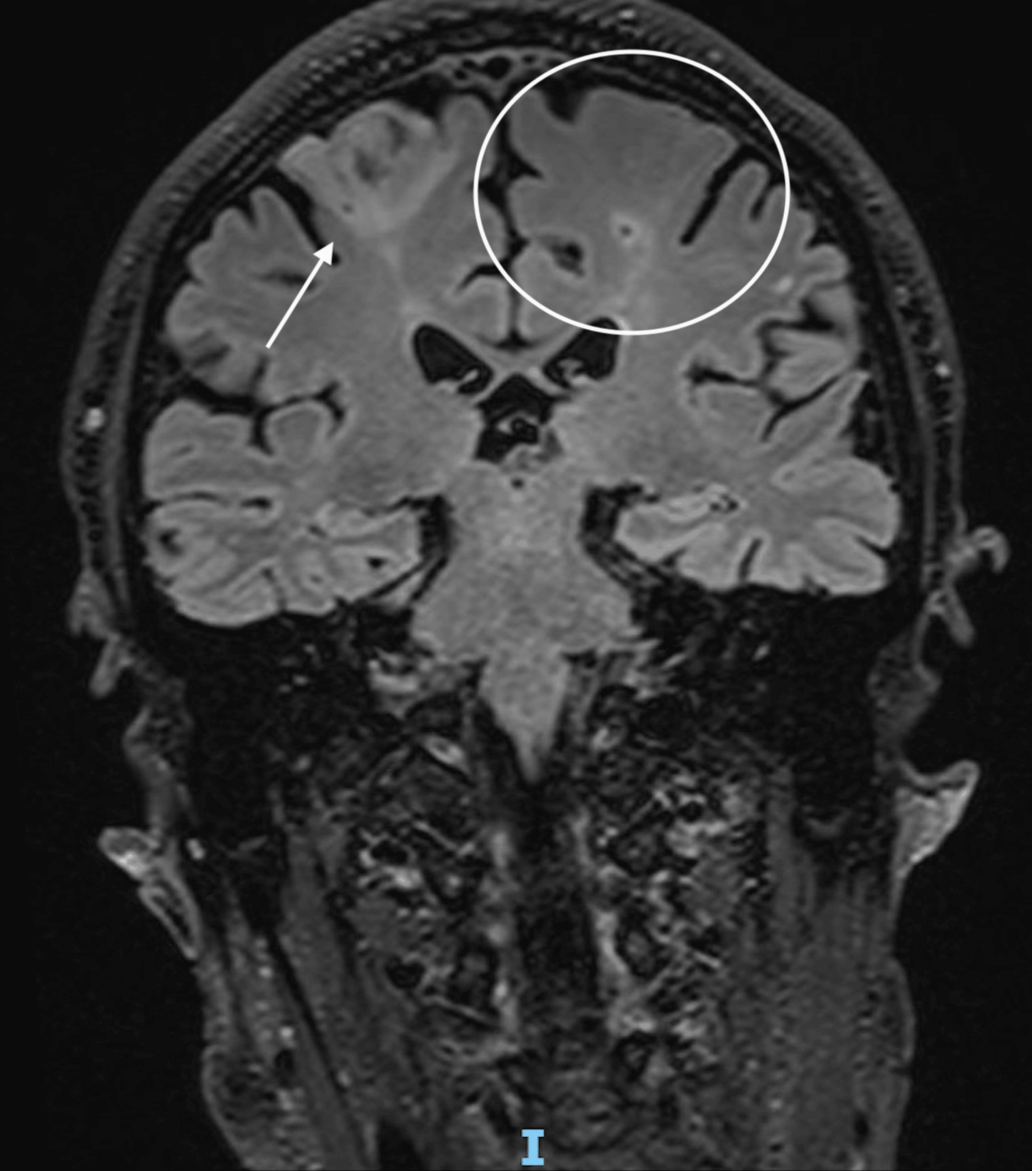
Brain CT showing bilateral evolving ischemic lesions

Considering the patient’s favorable clinical and laboratory evolution, with no fever and a decline in inflammatory markers, oral feeding was reintroduced on postoperative day 10. The next day, the patient developed chills, left hemiparesis, right hemiplegia, dysarthria, and confusion. Brain CT revealed bilateral evolving ischemic lesions (Figure 4).

The patient underwent repeat sternotomy under CPB. A perforation of the posterior wall of the left superior pulmonary vein was identified, sutured, and reinforced with a pericardial patch [5]. Esophageal sutures had dehisced, necessitating esophagectomy with left cervical esophagostomy.

Microbiological cultures from intraoperative samples and blood cultures grew *Streptococcus mitis*, *Neisseria macacae*, and *Staphylococcus epidermidis*. Antibiotic therapy was escalated to intravenous piperacillin-tazobactam and vancomycin for six weeks.

The patient achieved full neurological recovery and was discharged on postoperative day 43. Digestive tract reconstruction will be planned at a later stage.

## Discussion

This case illustrates the diagnostic and therapeutic challenges of AEF after AF ablation. Our patient initially presented without the classical triad of fever, neurological deficits, and gastrointestinal symptoms, as no atrial perforation was identified. The later occurrence of fever and neurological complications reflected the typical clinical course of AEF. Early resumption of oral feeding likely contributed to recurrence and esophageal suture dehiscence.

Chest CT with intravenous contrast is the gold-standard diagnostic tool [6]. Radiological findings may include pneumomediastinum, pericardial effusion, pneumopericardium, or direct visualization of an atrioesophageal communication. Brain CT can identify cerebral air embolism. In contrast, upper endoscopy and transesophageal echocardiography are contraindicated due to the risk of iatrogenic massive air embolism [7].

Surgical repair should be performed under CPB with aortic cross-clamping to minimize the risk of intraoperative embolization [8]. In this case, intraoperative endoscopy was safely performed under aortic cross-clamping. Postoperative management requires prolonged fasting and confirmation of esophageal healing by contrast esophagography before resuming oral intake.

## Conclusions

AEF after AF ablation is a rare but fatal complication requiring high clinical suspicion. Contrast-enhanced chest CT remains the diagnostic modality of choice. Surgical management under CPB with aortic cross-clamping is the recommended strategy. In this case, the premature advancement of dietary intake likely contributed to the recurrence. Delayed oral feeding, guided by radiologic evaluation such as esophagography, is essential to prevent recurrence.
